# To Treat or Not to Treat: Importance of Functional Dependence in Deciding Intravenous Thrombolysis of “Mild Stroke” Patients

**DOI:** 10.3390/jcm9030768

**Published:** 2020-03-12

**Authors:** Giovanni Merlino, Carmelo Smeralda, Simone Lorenzut, Gian Luigi Gigli, Andrea Surcinelli, Mariarosaria Valente

**Affiliations:** 1 Stroke Unit, Department of Neuroscience, Udine University Hospital, Piazzale S. Maria della Misericordia 15, 33100 Udine, Italy; simone.lorenzut@asufc.sanita.fvg.it; 2 Clinical Neurology, Udine University Hospital, 33100 Udine, Italy; carmelosmeralda@gmail.com (C.S.); gigli@uniud.it (G.L.G.); andsurcinelli@gmail.com (A.S.); mariarosaria.valente@uniud.it (M.V.); 3 Department of Medical Area (DAME), University of Udine, 33100 Udine, Italy; 4 Department of Mathematics, Informatics and Physics (DMIF), University of Udine, 33100 Udine, Italy

**Keywords:** intravenous thrombolysis, NIHSS, Barthel index, functional dependence

## Abstract

Intravenous thrombolysis (IVT) in patients with a low National Institutes of Health Stroke Scale (NIHSS) score of 0–5 remains controversial. IVT should be used in patients with mild but nevertheless disabling symptoms. We hypothesize that response to IVT of patients with “mild stroke” may depend on their level of functional dependence (FD) at hospital admission. The aims of our study were to investigate the effect of IVT and to explore the role of FD in influencing the response to IVT. This study was a retrospective analysis of a prospectively collected database, including 389 patients stratified into patients receiving IVT (IVT^+^) and not receiving IVT (IVT ^−^) just because of mild symptoms. Barthel index (BI) at admission was used to assess FD, dividing subjects with BI score < 80 (FD^+^) and with BI score ≥ 80 (FD^−^). The efficacy endpoints were the rate of positive disability outcome (DO^+^) (3-month mRS score of 0 or 1), and the rate of positive functional outcome (FO^+^) (mRS score of zero or one, plus BI score of 95 or 100 at 3 months). At the multivariate analysis, IVT treatment was an independent predictor of DO^+^ (OR 3.12, 95% CI 1.34−7.27, *p* = 0.008) and FO^+^ (OR: 4.70, 95% CI 2.38−9.26, *p* = 0.001). However, FD^+^ IVT^+^ patients had a significantly higher prevalence of DO^+^ and FO^+^ than those FD^+^ IVT^–^. Differently, IVT treatment did not influence DO^+^ and FO^+^ in FD^–^ patients. In FD^+^ patients, IVT treatment represented the strongest independent predictor of DO^+^ (OR 6.01, 95% CI 2.59–13.92, *p* = 0.001) and FO^+^ (OR 4.73, 95% CI 2.29–9.76, *p* = 0.001). In conclusion, alteplase seems to improve functional outcome in patients with “mild stroke”. However, in our experience, this beneficial effect is strongly influenced by FD at admission.

## 1. Introduction

Many patients with acute ischemic strokes (AIS) have a low National Institutes of Health Stroke Scale (NIHSS) score at presentation [1,2]. Although the presence of these mild symptoms represents the most common reason for renouncing intravenous thrombolysis (IVT) [3], only 68% of these patients can be discharged home without a residual disability [4]. Thus, there is increasing interest in the use of IVT in AIS patients with a low NIHSS score at admission. Results coming from clinical studies on this topic are conflicting, since functional outcome results, as assessed by the modified Rankin scale (mRS) sometimes improve, and at other times, are not modified by IVT treatment [5,6,7,8,9,10].

Previous American Heart Association/American Stroke Association guidelines suggested to use IVT treatment in persons with a *wide spectrum* of neurological deficits (1996) and with *measurable* neurological deficits (2007) [11,12]. This concept has been updated in the most version of the guidelines, recommending that IVT should also be used in patients with mild, but nevertheless disabling symptoms [13]. However, the NIHSS is not able to assess severity of disability. For instance, it cannot be used to accurately assess posterior circulation disease, which may cause very disabling symptoms. In fact, already in 2013, Wendt et al. reported that language impairment, distal paresis, and gait disorder were common disabling deficits in patients with low NIHSS scores. The authors suggest that the judgment of whether a stroke is disabling should not be based on the NIHSS score, but on the assessment of individual neurologic deficits and their impact on functional impairment [14].

To date, only a trial has been performed to compare the efficacy of alteplase versus aspirin for AIS patients with minor and non-disabling neurological deficits (the PRISMS trial). The authors observed that alteplase did not increase the likelihood of favorable outcome compared to aspirin [15,16]. Although the PRISMS was a prospective, double-blind, and placebo-controlled trial, it suffered from two significant limitations. In fact, the study was terminated early because patient recruitment was below target and it adopted a definition of “not clearly disabling” that was subjective and required interpretation by individual clinicians. Thus, conclusions of the PRISMS trial cannot be generalized.

A possible reason for the uncertain effectiveness of alteplase in minor strokes is that patients with a low NIHSS score at admission may respond to IVT treatment in different ways, depending on their level of functional dependence (FD) at admission. In addition, we suggest that the severity of FD should be assessed by a standard measure, such as the Barthel index (BI), instead of using a subjective selection based on the judgment of each physician. The aims of our study were: (1) to investigate the effects of IVT in patients with “mild stroke”, defined as a NIHSS score of 0−5 at presentation; (2) to explore the role of FD in influencing response to IVT in AIS patients with “mild stroke”.

## 2. Materials and Methods

### 2.1. Patients

This study was based on a retrospective analysis of a prospectively-collected database of consecutive patients admitted to the Udine University Hospital for AIS from January 2015 to December 2018. Inclusion criteria were: age 18 years or older and NIHSS score of 0 to 5. Exclusion criteria were: presence of a pre-stroke mRS score > 1, large vessel occlusion on cranial CT-angiography, and time interval > 4.5 h from symptoms onset. Out of 1636 patients admitted for AIS, 389 were considered suitable for the study after considering inclusion and exclusion criteria. The study sample was stratified into 2 groups: AIS patients who received IVT (IVT^+^) and patients to whom IVT was denied because of mild symptoms (IVT^−^).

### 2.2. Data Collection

The following variables were collected: age, sex, vascular risk factors such as previous transient ischemic attack or previous stroke, ischemic heart disease, peripheral artery disease, obesity defined as a BMI ≥ 30, atrial fibrillation, hypertension, diabetes mellitus, hypercholesterolemia, current smoking status, and pharmacological treatment. Stroke severity was determined with the NIHSS at admission. Presence of intracranial hemorrhage (ICH) was detected. Definition of symptomatic ICH (sICH) was based on the European Cooperative Acute Stroke Study (ECASS) III protocol [17]. Functional outcome was assessed by means of the mRS score 3 months after the stroke, and of the BI score, calculated at admission and recalculated at 3-months. The mRS and the BI scores after discharge were recorded at the patients’ routine clinical visit during a face-to-face examination.

### 2.3. Outcome Measures

Our efficacy endpoints were: (1) rate of positive disability outcome (DO^+^), defined as a 3-month mRS score of 0 or 1; (2) rate of positive functional outcome (FO^+^), defined as an mRS score of 0 or 1 *plus* a BI score of 95 or 100 at 3 months. The safety endpoints were: (1) rate of mortality at 3 months; (2) presence of sICH.

### 2.4. Statistical Analysis

Baseline characteristics and outcomes of the two patient groups (IVT^+^ versus IVT^−^) were compared by means of the chi-square test (Fisher’s exact test) for categorical variables and the Student’s *t*-test for independent samples when the continuous variables had a normal distribution.

The Mann–Whitney U test was used when the continuous variables had an abnormal distribution and for ordinal variables. Binary logistic regression was used to explore variables associated with outcome measures.

In order to explore whether there was a significant interaction between the types of presenting symptoms (according to the Barthel index) and the efficacy of thrombolysis, both patients treated and not treated with IVT were differentiated as: (1) patients without FD; (2) patients with FD predominantly due to weakness; (3) patients with FD predominantly due to imbalance; (4) patients with FD predominantly due to neglect and/or hemianopsia; (5) patients with FD predominantly due to other neurological symptoms, e.g., aphasia and confusion.

With the aim to verify if the level of FD at admission might influence response to IVT in AIS patients with “mild stroke”, we divided our sample into subjects with a BI score < 80 (FD^+^) and those with a BI score ≥ 80 (FD^−^). We tested this hypothesis comparing the efficacy endpoints between FD^+^ and FD^–^ patients, treated and not treated with IVT.

Data are displayed in tables as means and standard deviations (SD), if not otherwise specified. All probability values are two-tailed. A *p* value of < 0.05 was considered to be statistically significant. Statistical analysis was carried out using the SPSS Statistics, Version 20.0 for Windows (Chicago, IL, USA).

## 3. Results

**Figure 1:**
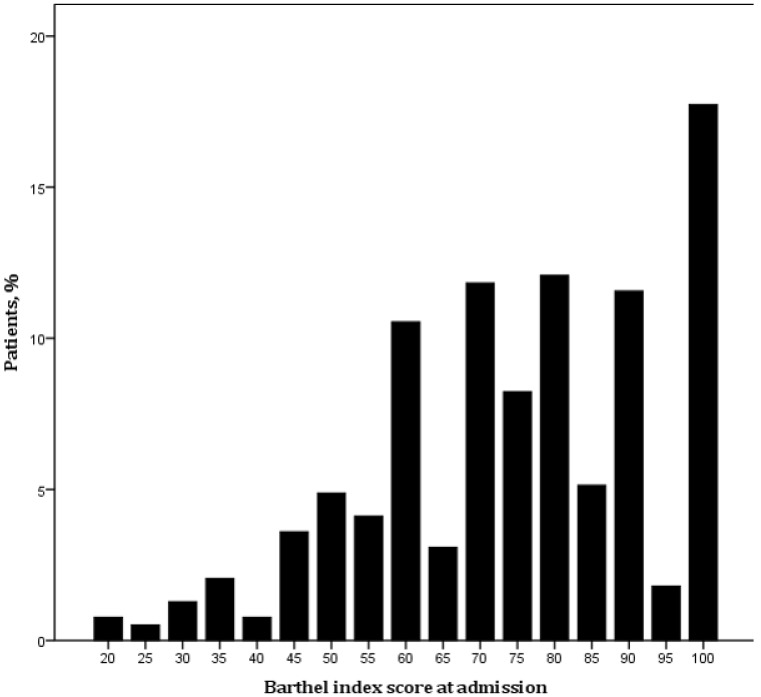
Barthel index score distribution in our sample.

Our sample of 389 patients was composed of 235 males (60.4%) with a mean age of 68.5 ± 13.6 years, a median NIHSS score of 2 (IQR 1−3), and a median BI score of 75 (IQR 60-90). Almost one-half (51.7%) of our patients with “mild stroke” had a BI score < 80 at admission (FD^+^ patients). Figure 1 shows the distribution of the BI score at admission in our sample.

Of the 389 enrolled patients with “mild stroke”, 113 (29%) were treated with IVT (IVT^+^), whereas in 276 (71%), IVT was denied because of mild symptoms (IVT^−^). Baseline characteristics in IVT^+^ and IVT^–^ patients are summarized in Table 1. The two groups differed only in median NIHSS score and use of anticoagulants.

At 3-months, IVT treatment improved both measures of outcome (DO^+^ and FO^+^). Although IVT^+^ patients showed higher rates of sICH than those IVT^–^, the prevalence of 3-month mortality did not differ between the two groups (see Table 2).

By univariate analysis, apart from IVT treatment, a positive disability outcome (DO^+^) was also associated with younger age (OR for 1-year increment in age 0.98, 95% CI 0.96–0.99, *p* = 0.02), lower NIHSS score at admission (OR 0.72 for 1-point increase in the scale, 95% CI 0.60–0.85, *p* = 0.001), higher BI score at admission (OR 1.06, 95% CI 1.05–1.08, *p* = 0.001), lower serum glucose at admission (OR 0.98 for each mg/dl increase in glucose, 95% CI 0.97–0.99, *p* = 0.01), a history of previous transient ischemic attack/stroke (OR 0.42, 95% CI 0.24–0.75, *p* = 0.003), obesity (OR 0.41, 95% CI 0.19–0.91, *p* = 0.02), and of diabetes mellitus (OR 0.45, 95% CI 0.27–0.77, p = 0.003). Regarding instead positive functional outcome (FO^+^), younger age (OR for 1-year increment in age 0.98, 95% CI 0.96–0.99, *p* = 0.008), lower NIHSS score at admission (OR 0.72 for 1-point increase in the scale, 95% CI 0.61–0.85, *p* = 0.001), higher BI score at admission (OR 1.06, 95% CI 1.04–1.07, *p* = 0.001), a history of previous transient ischemic attack/stroke (OR 0.45, 95% CI 0.26–0.80, *p* = 0.005), hypertension (OR 0.61, 95% CI 0.38–0.98, *p* = 0.04), and diabetes mellitus (OR 0.52, 95% CI 0.31–0.87, *p* = 0.01) were related to FO^+^ at 3 months.

By multivariate analysis, after controlling for variables significantly associated with the two efficacy endpoints at the univariate analysis, IVT treatment remained an independent predictor of DO^+^ and FO^+^ in patients with “mild stroke” (see Table 3).

A beneficial effect of IVT was observed only in patients with FD predominantly due to weakness who significantly improved after treatment (DO^+^: OR 4.88, 95% CI 2.01–11.83, *p* = 0.001; FO^+^: OR 5.03, 95% CI 2.23–11.32, *p* = 0.001), different from patients without FD, or with other types of presenting symptoms (data not shown).

**Figure 2:**
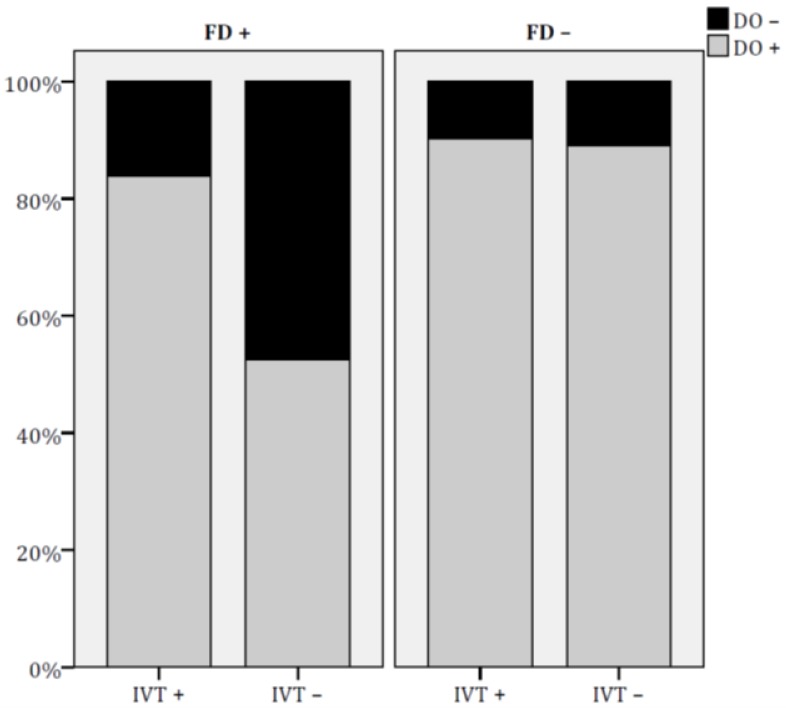
Effect of IVT on disability outcome rates in groups of patients with different levels of functional dependence at admission. DO = disability outcome; FD = functional dependence; IVT = intravenous thrombolysis.

**Figure 3:**
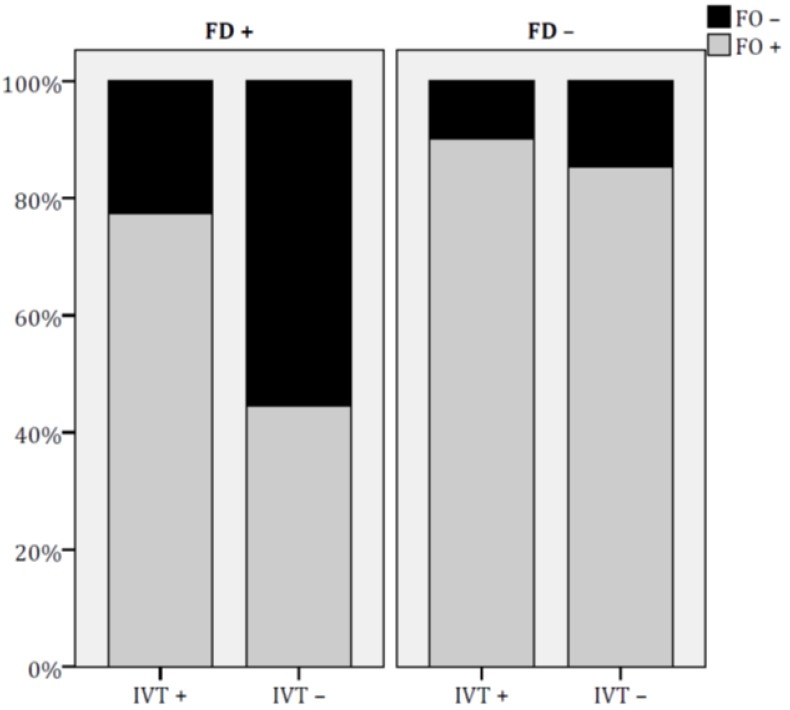
Effect of IVT on functional outcome rates in groups of patients with different levels of functional dependence at admission. FO= functional outcome; FD = functional dependence; IVT = intravenous thrombolysis.

As shown in Figure 2, the prevalence of DO^+^ was significantly higher in FD^+^ IVT^+^ patients than in those FD^+^ IVT ^–^ (FD^+^ IVT^+^: 83.9% vs. FD^+^ IVT^–^: 52.5%, *p* = 0.001); differently, IVT treatment did not influence DO^+^ in FD^–^ patients (FD^–^ IVT^+^: 90.2% vs. FD^–^ IVT^–^: 89.1%, p = 0.8). Similarly, for functional outcome, FO^+^ was significantly more common in FD^+^ IVT^+^ patients than in those FD^+^ IVT^–^ (FD^+^ IVT^+^: 77.4% vs. FD^+^ IVT^–^: 44.6%, *p* = 0.001), whereas rates of FO^+^ were similar in FD^–^ IVT^+^ and FD^–^ IVT^–^ patients (90.2% vs. 85.4%, *p* = 0.4) (see Figure 3).

In FD^+^ patients, the following variables were independent predictors of outcome: IVT treatment (OR 6.01, 95% CI 2.59–13.92, *p* = 0.001), BI score at admission (OR 1.07, 95% CI 1.04–1.10, *p* = 0.001), a history of previous transient ischemic attack/stroke (OR 0.41, 95% CI 0.18–0.92, *p* = 0.03), and diabetes mellitus (OR 0.42, 95% CI 0.18–0.94, *p* = 0.04) for DO^+^; IVT treatment (OR 4.73, 95% CI 2.29–9.76, *p* = 0.001), BI score at admission (OR 1.04, 95% CI 1.02–1.07, *p* = 0.001), and a history of previous transient ischemic attack/stroke (OR 0.44, 95% CI 0.20–0.96, *p* = 0.04) for FO^+^. In contrast, IVT treatment did not affect functional outcome in FD^–^ patients; in fact, BI score at admission was the only independent predictor of DO^+^ (OR 1.22, 95% CI 1.07–1.39, *p* = 0.002), and FO^+^ (OR 1.13, 95% CI 1.05–1.21, *p* = 0.001).

## 4. Discussion

For the first time we demonstrated that patients with “mild stroke”, as defined as a NIHSS score of 0−5, should be selected for IVT on the basis of their level of FD at admission. In particular, subjects with moderate or severe FD, as assessed by the BI score, should be treated with IVT as soon as possible. In contrast, treatment with alteplase seems to be ineffective on patients who are functionally independent or with slight FD at admission. Thus, our study gives support to the latest American Heart Association/American Stroke Association guidelines recommending that IVT should be used for patients with mild but also disabling symptoms [13].

There is ongoing debate concerning what is a “mild stroke”. In 2010, Fisher et al. explored the relationship of 6 different “minor stroke” definitions and outcomes. Since patients with a NIHSS score ≤ 3 had the best short- and medium-term outcome, the authors suggested to use this easily-applicable definition [18]. Although this definition has been used in some studies [19,20], a recent review of this topic reported that the NIHSS—with a score ranging from 0 to 5—is the most commonly used tool to define a “mild stroke” [21]. Similarly, we utilized an NIHSS score of 0 to 5 for identifying patients with supposed “mild” symptoms; our patients had a median NIHSS score of 2. However, despite their low NIHSS, several of them were affected by severe FD at admission; in fact, we observed a median BI score of 75, and 51.7% of the sample had a BI score < 80. This discrepancy may be due to the fact that the NIHSS is not able to detect symptoms of posterior circulation stroke, such as postural instability, gait disturbance, and dysphagia that can cause very severe disability. Thus, we think that patients with a low NIHSS score at presentation should be carefully evaluated regarding the presence of possible disabling symptoms before being labeled as affected by “mild stroke”.

Originally published in 1965, the BI was developed to give physicians a suitable standard tool to assess and measure FD [22]. In fact, the BI covers all activities considered part of any assessment of activities of daily living, has an excellent reliability and validity, is easy to use, and only takes a few minutes [23]. Thus, we suggest adopting this tool in patients with “mild stroke”, in order to correctly recognize patients with non-disabling symptoms.

Obviously, the exact distinction between stroke with disabling or non-disabling symptoms becomes extremely important when patients are affected by AIS and are suitable for IVT treatment. In our sample, more than 70% of AIS patients were not treated with IVT because they were deemed too good to be treated. This rate is perfectly in line with previous studies on this topic [6,9,10]. As shown in Table 1, the decision to treat or not to treat with IVT was merely based on the NIHSS score, whereas the level of FD was absolutely neglected. Use of anticoagulant agents was, as expected, significantly higher in AIS patients with “mild stroke” who were not treated, than in those who received IVT.

Previous studies on IVT treatment in AIS patients with mild symptoms report conflicting results [5,6,7,8,9,10]. In 2012 Huisa et al. investigated 133 patients with minor ischemic strokes, defined as an admission NIHSS score ≤ 5, and observed similar outcomes between patients treated and not treated with alteplase [5]. An Italian study of 128 patients with mild ischemic stroke confirmed that alteplase did not improve functional outcome [6]. Of 276 patients with mild ischemic stroke symptoms that were analyzed by Spokoyny et al., 83 were IVT treated. Treated and untreated patients had similar baseline characteristics except that the treated group had higher baseline NIHSS. Prevalence of mRS 2−6 at 90 days was 37.4% in the treated group and 31.1% in the untreated group (*p* = 0.44) [9]. In contrast, Urra et al. reported that IVT was associated with a greater proportion of patients with mild stroke who shifted down on the mRS score at 3 months (OR 2.66; 95% CI 1.49–4.74, *p* = 0.001) [7]. In a case-control study of 890 Austrian patients with a NIHSS score 0−5 at admission, IVT was associated with a better outcome after 3 months (OR 1.49, 95% CI 1.17–1.89, *p* < 0.001) [8]. More recently, Haeberlin et al. compared 3-month functional outcomes in 370 consecutive AIS patients with a NIHSS score ≤ 6. Although patients with mild AIS had a high chance of favorable outcomes irrespective of treatment type, subjects receiving IVT more often achieved complete remission of symptoms (mRS score = 0) (OR 3.33, *p* < 0.0001) [10]. Similarly to Urra et al. [7] and Haeberlin et al. [10], we observed a major beneficial effect of IVT on the outcome measures. Interestingly, it would seem that IVT efficacy is more pronounced in AIS patients affected by “mild stroke” than in those enrolled in regulatory randomized controlled trials, in which patients with minor symptoms were largely underrepresented [24]. We think that this discrepancy may be due to different study design between observational studies and randomized controlled trials. In fact, more often, non-interventional studies tend to overestimate the effects of the treatment and show more variability in estimates of the effects because of residual confounding, errors, and bias.

Discording results of IVT efficacy in “mild strokes” may be explained by differences in clinical characteristics among patients with minor symptoms. In particular, our patients who underwent IVT had a better 3-month functional outcome than those IVT^–^, but presence of neurological symptoms due to weakness and level of FD at admission played a major role in influencing this association. If patients with “mild stroke” *plus* disabling symptoms (FD^+^) were treated with alteplase, there was a significant improvement in functional outcome compared to those which were not treated. Indeed, more than 50% of patients with a BI score < 80 for whom IVT was denied did not achieve functional independence 3 months after stroke. In patients with “mild stroke” *plus* disabling symptoms, IVT represented the strongest predictor of DO^+^ (OR: 6.01) and FO^+^ (OR: 4.73). On the other hand, rates of favorable outcomes were very high in patients without disability, regardless of treatment type. In these patients, BI score at admission was the only independent predictor of DO^+^ and FO^+^, while IVT treatment did not influence functional outcomes. In contrast, Urra et al. report that IVT was associated with a greater proportion of patients with non-disabling minor strokes who shifted down on the mRS score at 3-months [7].

To date, only the PRISMS trial has been designed to assess the efficacy of IVT for the treatment of AIS with NIHSS 0−5, and without clearly disabling deficits. The authors designed a multicenter, randomized, double-blind, placebo-controlled trial with a sample size of 948 subjects [15]. Unfortunately, the study was terminated early because of low patient recruitment. Results of the 313 patients enrolled failed to demonstrate more favorable functional outcomes in patients treated with alteplase, as compared to those receiving only aspirin. However, the trial’s early termination precludes any definitive conclusions on this topic. Moreover, definition of “not clearly disabling” was left to the subjective interpretation of individual clinicians [16].

Regarding safety endpoints, in our sample, alteplase treatment significantly increased the risk of sICH, even if rates of mortality were similar in patients IVT^+^ and IVT^–^. Bearing in mind that higher NIHSS scores predict a higher rate of sICH, it could be argued that our sICH rate in patients with “mild stroke” was high. However, a previous study performed in patients with minor stroke reported a sICH rate as high as 5% when IVT was administered [5].

Several limitations of this study need to be acknowledged. First, the retrospective design of our study was certainly a limit; however, all data were prospectively collected. Second, measures of outcome were obtained by physicians that were not blinded to IVT treatment, which may have influenced their rating. Third, information on intervals between stroke onset and IVT was not collected, thus we cannot exclude that elapsed time from symptoms onset may have influenced physicians’ decisions to perform or not perform IVT treatment. Finally, since this was a hypothesis-generating study, further surveys are needed to test our preliminary hypotheses. In particular, interventional trials should be performed in order to exclude the presence of a bias by indication that could have affected our observational study.

In conclusion, alteplase seems to improve functional outcome in patients with a low NIHSS score. However, in our experience, this beneficial effect is strongly influenced by FD at admission. In patients with “mild stroke” *plus* disabling symptoms, IVT treatment should be administered as soon as possible; on the contrary, alteplase may not be used if minor and non-disabling deficits are diagnosed. In order to distinguish mild ischemic stroke patients with disabling or non-disabling symptoms, we suggest to use the BI. Our observational study brings further evidence to the results coming from a few other non-interventional studies and from one randomized trial interrupted before completion. Thus, further large interventional studies are needed to confirm our preliminary findings.

## Figures and Tables

**Table 1:** Baseline characteristics.

	IVT^+^ (n = 113)	IVT^–^ (n = 276)	*p*
**Demographic data and baseline clinical characteristics**			
Age, years	68.2 ± 12.0	68.7 ± 14.2	0.7
Males, n (%)	67 (59.3)	168 (60.9)	0.8
NIHSS score at admission, median (IQR)	3 (2−4)	2 (1−3)	0.001
BI score at admission, median (IQR)	60 (75−90)	60 (75−90)	0.5
Medications prior to onset, n (%)			
Antiplatelet agents	39 (34.5)	79 (28.6)	0.2
Anticoagulant agents	1 (0.9)	25 (9.1)	0.003
Glucose level, mg/dl	126.4 ± 35.3	134.2 ± 64.7	0.3
**Vascular risk factors**			
Previous transient ischemic attack/stroke, n (%)	15 (13.3)	45 (16.3)	0.4
Ischemic heart disease, n (%)	19 (18.3)	27 (10.8)	0.06
Peripheral artery disease, n (%)	2 (1.9)	2 (0.8)	0.6
Obesity, n (%)	9 (8.7)	22 (8.8)	0.9
Atrial fibrillation, n (%)	19 (16.8)	67 (24.3)	0.1
Hypertension, n (%)	68 (60.2)	184 (66.7)	0.2
Diabetes mellitus, n (%)	17 (15.0)	63 (22.8)	0.08
Hypercholesterolemia, n (%)	43 (38.1)	102 (37.0)	0.8
Current smoking, n (%)	29 (26.1)	53 (21.1)	0.3

IVT = intravenous thrombolysis; NIHSS = National Institute of Health Stroke scale; BI = Barthel index.

**Table 2:** Efficacy and safety endpoints in patients treated and not treated with IVT.

	IVT^+^ (n = 113)	IVT^–^ (n = 276)	*p*
**Efficacy endpoints**			
DO^+^, n (%)	98 (86.7)	195 (70.7)	0.001
FO^+^, n (%)	94 (83.9)	179 (65.6)	0.001
**Safety endpoints**			
Mortality at 3-months, n (%)	1 (0.9)	3 (1.1)	0.8
sICH, n (%)	5 (4.4)	3 (1.1)	0.03

IVT = intravenous thrombolysis; DO^+^ (3-month mRS score of 0 or 1) = positive disability outcome; FO^+^ (mRS score of 0 or 1, *plus* BI score of 95 or 100 at 3-months) = positive functional outcome; sICH = symptomatic intracranial hemorrhage.

**Table 3:** Multivariable analyses showing independent predictors of positive disability outcome and positive functional outcome.

**DO^+^**	**OR**	**95% CI**	***p***
IVT treatment			
No	1.00		
Yes	3.12	1.34−7.27	0.008
Age	0.95	0.92−0.98	0.003
BI score at admission	1.06	1.04−1.09	0.001
**FO^+^**	**OR**	**95% CI**	***p***
IVT treatment			
No	1.00		
Yes	4.70	2.38−9.26	0.001
Age	0.98	0.96−0.99	0.02
NIHSS score at admission	0.77	0.61−0.97	0.03
BI score at admission	1.06	1.04−1.07	0.001
Diabetes mellitus			
No	1.00		
Yes	0.53	0.29−0.98	0.04

DO^+^ (3-month mRS score of 0 or 1) = positive disability outcome; FO^+^ (mRS score of 0 or 1 *plus* BI score of 95 or 100 at 3-months) = positive functional outcome; IVT = intravenous thrombolysis; NIHSS = National Institute of Health Stroke scale; BI = Barthel index.

## References

[1] Reeves M., Khoury J., Alwell K., Moomaw C., Flaherty M., Woo D., Khatri P., Adeoye O., Ferioli S., Kissela N. (2013). Distribution of national institutes of health stroke scale in the Cincinnati/Northern Kentucky stroke study. Stroke.

[2] Dhamoon M.S., Moon Y.P., Paik M.C., Boden-Albala B., Rundek T., Sacco R.L., Elkind M.S. (2009). Long-term functional recovery after first ischemic stroke. Stroke.

[3] Messé S.R., Khatri P., Reeves M.J., Smith E.E., Saver J.L., Bhatt D.L., Grau-Sepulveda M.V., Cox M., Peterson E.D., Fonarow G.C. (2016). Why are acute ischemic stroke patients not receiving IV tPA? Results from a national registry. Neurology.

[4] Barber P.A., Zhang J., Demchuk A.M., Hill M.D., Buchan A.M. (2001). Why are stroke patients excluded from TPA therapy? An analysis of patient eligibility. Neurology.

[5] Huisa B.N., Raman R., Neil W., Ernstrom K., Hemmen T. (2012). Intravenous tissue plasminogen activator for patients with minor ischemic stroke. J. Stroke Cerebrovasc. Dis..

[6] Nesi M., Lucente G., Nencini P., Fancellu L., Inzitari D. (2014). Aphasia predicts unfavorable outcome in mild ischemic stroke patients and prompts thrombolytic treatment. J. Stroke Cerebrovasc. Dis..

[7] Urra X., Ariño H., Llull L., Amaro S., Obach V., Cervera A., Chamorro A. (2013). The outcome of patients with mild stroke improves after treatment with systemic thrombolysis. PLoS ONE.

[8] Greisenegger S., Seyfang L., Kiechl S., Lang W., Ferrari J. (2014). Austrian Stroke Unit Registry Collaborators. Thrombolysis in patients with mild stroke: Results from the Austrian Stroke Unit Registry. Stroke.

[9] Spokoyny I., Raman R., Ernstrom K., Khatri P., Meyer D.M., Hemmen T.M., Meyer B.C. (2015). Defining mild stroke: Outcomes analysis of treated and untreated mild stroke patients. J. Stroke Cerebrovasc. Dis..

[10] Haeberlin M.I., Held U., Baumgartner R.W., Georgiadis D., Valko P.O. (2019). Impact of intravenous thrombolysis on functional outcome in patients with mild ischemic stroke without large vessel occlusion or rapidly improving symptoms. Int. J. Stroke.

[11] Adams H.P., Brott T.G., Furlan A.J., Gomez C.R., Grotta J., Helgason C.M., Kwiatkowski T., Lyden P.D., Marler J.R., Torner J. (1996). Guidelines for thrombolytic therapy for acute stroke: A supplement to the guidelines for the management of patients with acute ischemic stroke. A statement for healthcare professionals from a special writing group of the Stroke Council, American Heart Association. Stroke.

[12] Adams H.O., del Zoppo G., Alberts M.J., Bhatt D.L., Brass L., Furlan A., Grubb R.L., Higashida R.T., Jauch E.C., Kidwell C. (2007). American Heart Association, American Stroke Association Stroke Council, Clinical Cardiology Council, Cardiovascular Radiology and Intervention Council, Atherosclerotic Peripheral Vascular Disease and Quality of Care Outcomes in Research Interdisciplinary Working Groups. Guidelines for the early management of adults with ischemic stroke: A guideline from the American Heart Association/American Stroke Association Stroke Council, Clinical Cardiology Council, Cardiovascular Radiology and Intervention Council, and the Atherosclerotic Peripheral Vascular Disease and Quality of Care Outcomes in Research Interdisciplinary Working Groups: The American Academy of Neurology Affirms the Value of This Guideline as an Educational Tool for Neurologists. Stroke.

[13] Powers W.J., Rabinstein A.A., Ackerson T., Adeoye O.M., Bambakidis N.C., Becker K., Biller J., Brown M., Demaerschalk B.M., David L. (2018). American Heart Association Stroke Council. 2018 guidelines for the early management of patients with acute ischemic stroke: A guideline for healthcare professionals from the American Heart Association/American Stroke Association. Stroke.

[14] Wendt M., Tutuncu S., Fiebach J.B., Scheitz J.F., Audebert H.J., Nolte C.H. (2013). Preclusion of ischemic stroke patients from intravenous tissue plasminogen activator treatment for mild symptoms should not be based on low National Institutes of Health Stroke Scale scores. J. Stroke Cerebrovasc. Dis..

[15] Yeatts S.D., Broderick J.P., Chatterjee A., Jauch E.C., Levine S.R., Romano J.G., Saver J.L., Vagal A., Purdon B., Devenport J. (2018). Alteplase for the treatment of acute ischemic stroke in patients with low National Institutes of Health Stroke Scale and not clearly disabling deficits (Potential of rtPA for Ischemic Strokes with Mild Symptoms PRISMS): Rationale and design. Int. J. Stroke.

[16] Khatri P., Kleindorfer D.O., Devlin T., Sawyer R.N., Starr M., Mejilla J., Broderick J., Chatterjee A., Jauch E.C., Levine S.R. (2018). Effect of alteplase vs. aspirin on functional outcome for patients with acute ischemic stroke and minor nondisabling neur logic deficits: The PRISMS randomized clinical trial. JAMA.

[17] Hacke W., Kaste M., Bluhmki E., Brozman M., Davalos A., Guidetti D., Larrue V., Lees K.R., Medeghri Z., Machnig T. (2008). Thrombolysis with alteplase 3 to 4.5 h after acute ischemic stroke. N. Engl. J. Med..

[18] Fischer U., Baumgartner A., Arnold M., Nedeltchev K., Gralla J., Marco De Marchis G., Kappeler L., Mono M.-L., Brekenfeld C., Schroth G. (2010). What is a minor stroke?. Stroke.

[19] Luengo-Fernandez R., Gray A.M., Rothwell P.M. (2009). Effect of urgent treatment for transient ischaemic attack and minor stroke on disability and hospital costs (EXPRESS study): A prospective population-based sequential comparison. Lancet Neurol..

[20] Coutts S.B., Hill M.D., Campos C.R., Choi Y.B., Subramaniam S., Kosior J.C., Demchuk A.M. (2008). Recurrent events in transient ischemic attack and minor stroke. Stroke.

[21] Schwartz J.K., Capo-Lugo C.E., Akinwuntan A.E., Roberts P., Krishnan S., Belagaje S.R., Lovic M., Burns S.P., Hu X., Danzl M. (2019). Classification of mild stroke: A mapping review. Pm&r.

[22] Mahoney F.I., Barthel D.W. (1965). Functional evaluation: The Barthel index. Md. State Med. J..

[23] Barak S., Duncan P.W. (2006). Issues in selecting outcome measures to assess functional recovery after stroke. NeuroRx.

[24] Wardlaw Wardlaw J.M., Murray V., Berge E., del Zoppo G.J. (2014). Thrombolysis for acute ischemic stroke. Cochrane Database Syst. Rev..

